# Population-level impact of loss on survivor mortality risk

**DOI:** 10.1007/s11136-015-1048-x

**Published:** 2015-06-17

**Authors:** Joseph Allegra, Amara Ezeamama, Cherie Simpson, Toni Miles

**Affiliations:** University of Georgia Athens, Athens, GA USA

**Keywords:** Population-level health, Bereavement, Longevity

## Abstract

**Introduction:**

The loss of a loved one adversely affects the bereaved.

**Materials and Methods:**

Using data from the 2010 and 2012 waves of Health and Retirement Study (HRS), we estimate the risk for death in a 2-year span after the loss of a parent, spouse, or child for adults aged 50 to 70 years.

**Conclusion:**

A respondent with a loss was twice as likely to die when compared similarly aged persons with no loss (OR 2.32; 95 % CI 1.14, 5.30). Loss of either a parent (OR 1.93; 95 % CI 1.01, 4.07), or a child (OR 1.77; 95 % CI 1.08, 2.96) also increased respondent mortality. This elevated risk persists after adjustment for gender and other high-risk health conditions. Any physical activity reduces survivor death rates during this critical period by more than 85 %.

## Introduction

The death of a parent or spouse adversely affects the bereaved [1, 2]. Likewise, the loss of a child affects the health of the parent(s) [3, 4]. As the population’s age increases, the overlap between the parent and child lifespan also increases. Eighty-five percent of individuals between the ages 40–50 years have a living parent [5]. Newly published reports link population-level insomnia and healthcare utilization to loss, as a potential risk factor for illness in the USA. In a cohort of men and women ages 50–70 years, loss increased the risk for clinical insomnia [6] and hospitalization rates by 20–30 % [7]. This risk persists after controlling for age, gender, and health and behavior status. In a longevity society, loss is an emerging public health issue.

## Methods

We estimated the risk that loss poses for a survivor’s subsequent mortality with data from the 2010 and 2012 wave of the Health and Retirement Study (HRS). HRS is a representative sample of Americans aged 50 years and older. For purposes of these analyses, and in congruence with our previous research [6, 7], our sample was limited to respondents aged 50–70 years in both the 2010 and 2012 waves of HRS (*N* = 5757).

The primary exposure in this study is reported death of a parent, spouse, sibling, or child. We categorized the death in three ways: any loss (yes or no), relationship between respondent and decedent, and the number of losses experienced by the respondent. The survey did not measure deaths outside of these relationships. The final generalized linear models did not include loss of a sibling due to low numbers of events (*N* = 2). We did retain these numbers when constructing a summary figure (total losses).

We assessed the mortality of the respondent using the HRS 2012 vital status variable. Seventy-five percent of respondents between the ages of 50 and 70 years survived between the end of the 2010 wave and the start of the 2012 wave.

In the model, age was categorical in five-year intervals, and we included a number of covariates. These potential confounders included gender, physical activity (any versus none), and health insurance (any versus none). We also included a measure of satisfaction with current health status as well as a four-level measure of insomnia symptoms.

We used Stata 12 (StataCorp, LP College Station, TX, USA) for all descriptive statistics, measures of association (odds ratios with 95 % confidence intervals), and generalized linear models using the logit link function $g\left( \mu \right) = \log [\frac{\mu }{1 - \mu }]$. The logit link is useful for binary outcomes and logistic regression. Because the outcome was rare, we determined that odds ratios were an appropriate measure of association and would approximate the relative risk.

## Results

The mortality rate increased twofold from the youngest to oldest age categories between survey waves. A respondent with a loss was twice as likely to die when compared similarly aged persons with no loss (OR 2.32; 95 % CI 1.14, 5.30). Loss of either a parent (OR 1.93; 95 % CI 1.01, 4.07), or a child (OR 1.77; 95 % CI 1.08, 2.96) also increased respondent mortality.

Table 1 shows the final models for each loss variable and indicates that loss of any type has a significant effect on the survivors own mortality. Moreover, multiple losses more than double the odds of survivor mortality. After controlling for gender, physical activity, and insomnia symptoms, the odds ratio for survivor mortality is 1.78 (95 % CI 1.01, 3.16) for 2 losses, and 3.84 (95 % CI 1.29, 11.41) for three or more losses. The results also suggest that any level of physical activity (mild, moderate, or vigorous) significantly reduces survivor death risk.

**Table 1:** Final generalized linear models for respondent mortality and loss

	Unadjusted OR (95 % CI)	Model I	Model II	Model III	Model IV	Model V	Model VI
Any loss	2.32 (1.14, 5.30)*	2.30 (1.14, 4.65)*					
Loss of parent	1.93 (1.01, 4.07)*		1.92 (1.00, 3.66)*				
Loss of spouse	1.94 (0.80, 4.09)			2.17 (1.01, 4.68)*			
Loss of child	1.77 (1.08, 2.96)*				1.78 (1.10, 2.88)*		
2 Losses	1.91 (1.02, 3.39)*					1.78 (1.01, 3.16)*	
3 +Losses	4.33 (1.12, 12.05)**						3.84 (1.29, 11.41)*
Gender^a^		1.96 (1.22, 3.14)**	1.94 (1.21, 3.11)**	1.90 (1.18, 3.06)**	2.03 (1.27, 3.26)**	1.99 (1.24, 3.19)**	2.02 (1.25, 3.24)*
Insomnia symptoms^b^		1.53 (1.10, 2.13)*	1.54 (1.10, 2.14)*		1.55 (1.11, 2.16)**	1.52 (1.10, 2.12)*	1.54 (1.11, 2.14)**
Physical activity^c^		0.14 (0.08, 0.25)**	0.13 (0.07, 0.25)**	0.12 (0.07, 0.22)**	0.15 (0.08, 0.26)**	0.14 (0.08, 0.26)**	0.14 (0.08, 0.27)***

Models I–VI adjusts for gender, physical activity, and insomnia symptoms (except model III) and predicts respondent mortality based on any loss (Model I), type of loss (Models II–IV), and number of losses (Models V and VI). The reference group for the models is respondents without a loss for the particular family member

^a^Reference group = female, ^b^ reference group = no insomnia symptoms, ^c^ reference group = no physical activity

* = *p* < 0.05; ** = *p* < 0.01

## Discussion

In a longevity society, parents and children can live together for more than 50 years. The new science of social network provides a mechanism for the propagation of illness caused by negative emotions [8]. Our results show that the loss of close relative (parent, spouse, sibling, or child) is an independent contributor to risk for mortality of the bereaved. These results are consistent with other population-level reports [1]. Our results also suggest that physical activity strongly reverses this risk. If loss does act as a gateway to healthcare system use as our prior research suggests, then the implementation of an activity regimen as part of a hospice bereavement benefit, could provide a measureable impact on population-level health care utilization.
